# Morphometrics of the Tropical Bed Bug (Hemiptera: Cimicidae) From Cape Coast, Ghana

**DOI:** 10.1093/jme/tjac072

**Published:** 2022-06-15

**Authors:** Godwin Deku, Rofela Combey, Stephen L Doggett

**Affiliations:** Department of Conservation Biology and Entomology, School of Biological Sciences, University of Cape Coast, Cape Coast, Ghana; Department of Conservation Biology and Entomology, School of Biological Sciences, University of Cape Coast, Cape Coast, Ghana; Department of Medical Entomology, NSW Health Pathology-ICPMR, Westmead Hospital, Sydney, Australia

**Keywords:** tropical bed bug, geometric morphometrics, subpopulation, nymph, Cape Coast

## Abstract

Bed bugs, *Cimex lectularius* (L.) (Hemiptera: Cimicidae) and *Cimex hemipterus* (F.), have become established worldwide in recent years largely due to the development of insecticide resistance. However, limited attention has been given to ongoing morphological and macroevolutionary changes within the species and their populations, which could have implications for their control. Here, we evaluated whether bed bugs of the species *C. hemipterus* inhabiting different communities in Cape Coast, Ghana are undergoing segregation, which could lead to possible speciation. We also aimed to provide a morphometric description of all nymphal stages. Nine-bed bug populations of *C. hemipterus* were field-collected in Cape Coast and were subjected to geometric morphometric analysis. The multivariate parameters applied distinguished various populations from each of the locations, indicating the presence of morphologically distinct subpopulations of *C. hemipterus*. Shape-based segregation and shape changes associated with the insect pronotum (which is an important taxonomic character in the Cimicidae) were evident across the populations. Through this comparative study of *C. hemipterus*, we showed that possible subpopulations of this bed bug are being spread from Ghana. The nymphal stages (first–fifth) of *C. hemipterus* were distinguished by the length of the last three antennal segment and pronota width; such information contributes to the taxonomic knowledge of the species.

Bed bugs are blood-sucking insects belonging to the family Cimicidae. *Cimex* species are widespread globally, and presently, 23 species of the genus have been described, although the taxonomic status of some of these species remain contentious (Akhoundi et al. 2020). In the *Monograph of the Cimicidae* (Usinger 1966), the 16 species of the genus listed are categorized into four groups that feed primarily on humans and bats, and occasionally birds. The Lectularius and Hemipterus groups are found predominantly in the Cosmopolitan and the Tropicopolitan regions, respectively, where they feed on humans, bats, and poultry. The *Pilosellus* and *Pipistrelli* group are found predominantly in the Nearctic and the Palearctic regions, respectively, and feed mainly on bats (Usinger 1966). Of the members of the family Cimicidae, only three species: *Leptocimex boueti* (Brumpt), *Cimex hemipterus* (F.), and *Cimex lectularius* (L.) are called ‘bed bugs’ due to their close association with humans (Newberry 1989). *Leptocimex boueti* is presently found in West Africa only (Usinger 1966). *Cimex hemipterus* is mostly found in the tropics of the Old and New Worlds and is the most widely distributed bed bug species in Africa and larger parts of Asia and India (Zorrilla Vaca et al. 2015). The species has also been more recently recorded in Australia (Doggett et al. 2003, Doggett and Cains 2018), Europe (Berenger et al. 2017, Naylor et al. 2018, Masini et al. 2020, Balvín et al. 2021), Russia (Gapon 2016, Golub et al. 2020, Martynov et al. 2021, Prisniy 2021), and the USA (Campbell et al. 2016, Lewis et al. 2020). *Cimex lectularius* occurs in temperate and subtropical regions worldwide (Newberry 1989, Schuh and Slater 1995, Eddy and Jones 2011) and is the main species occurring in Europe and America (Eddy and Jones 2011, Zorrilla Vaca et al. 2015). Comparisons of the two major bed bug species, *C. lectularius* and *C. hemipterus,* with emphasis on their distribution, biology, infestation rates, and control, have been extensively reviewed by several authors in the past (Mellanby 1935, Mellaby 1939, Ömori 1939, Johnson 1939, Lewis 1949, Busvine 1963, Davis 1964, Usinger 1966, Mekuria 1967, Newberry 1989). Although a review on bed bugs in Africa was recently published (Fourie and Crafford 2018), this article did not extensively include information on the morphology of the species. Over the last two decades, bed bugs (*C. hemipterus* and *C. lectularius*) have emerged globally, and the increase in bed bug numbers is largely thought to be related to the development of insecticide resistance, and the subsequent spread of these resistant strains through anthropocentric activities (Doggett et al. 2018).

The extent of the bed bug reemergence with its associated insecticide resistance problems have been evidenced in Africa, including reports coming from Tanzania, Senegal, Nigeria, Gambia, Kenya, Sierra Leone, Zimbabwe, and Ghana (Gbakima et al. 2002, Fourie and Crafford 2018, Deku et al. 2021, Ndiaye et al. 2022). These papers highlight the need to undertake a morphometric review on the major bed bug species involved in the resurgence as morphological variant populations can reveal the geographical origin of an insect pest (Schofield 2000). Enhanced taxonomic identification keys for species and all nymphal stages will be effectively useful in field epidemiological surveillance and monitoring.

Morphometrics is commonly considered as a tool for assessing phenotypic variation among species and can be used to detect local adaptations, population divergence (Dujardin and Slice 2007), and the characterization of a species (Combey et al. 2013). In the hemipterans, body shape has been reported to be an important diagnostic feature (Bouchard 2004). To date, morphological variations in body shape within the various bed bug species have not been analyzed according to morphometric techniques. Changes in body shape, or the shape of functional structures, have numerous ecological implications such as sexual selection, feeding, adaptation, aggressiveness, competition, reproduction and development, predation, and fitness (Alves and Hernández 2017). Furthermore, modification in general body shape is a recurring theme among some ectoparasitic insects, facilitating their movement on the host and enabling them to hide in tight spaces (Burkett-Cadena 2019). Beyond the taxonomic and the ecological implications of body shape modifications, morphology may also indicate the impact of environmental stressors on an insect population (Alves and Hernández 2017), which can have implications for physical, chemical, and mechanical control measures.

In a wingless and isolated insect population such as bed bugs that lack the ability to fly and frequently disperse to interact reproductively with a larger population, host and reproductive factors as well as environmental factors have the capacity to result in genetic, reproductive, morphological, and behavioral differentiation in the insect populations (Mikery et al. 2019). In related insect species such as the Phlebotominae (Diptera: Psychodidae), adaptive plasticity, wide distribution, limited dispersal, and reproductive activity have resulted in genetic, morphometric, and reproductive variations within populations of the species (Florin et al. 2011). These processes result in sympatric speciation within the species population that are not distinguishable by classical taxonomy, which fails to recognize sibling and cryptic species (Mikery et al. 2019).

In bed bugs, sexual conflicts occur and sympatric speciation from sexual conflicts have been theoretically proven in species populations (Engen and Bernt-Erik 1985, Gavrilets and Waxman 2002). Sexual conflict is when the evolutionary interest of males and females diverge and can manifest in two ways, which includes ‘Interlocus’ (optimal outcome of male and female interactions differs between the two sexes) and ‘Intralocus’ (a trait expressed in both sexes has opposite effects on male and female fitness), with both processes resulting in coevolution and genetic variation (Pischedda and Stewart 2016). In the model by Gavrilets and Waxman (2002), two general regimes were developed. First, instead of females running away to avoiding males from mating, females can diversify genetically into separate groups. The males may then respond to the female’s diversification by diversifying themselves. This continuous coevolutionary process results in the formation of reproductively isolated clusters of genotypes that emerge into diverged populations. Furthermore, the host on which an organism feeds can also contribute to divergence in the species. Recently, some researchers found that *C. lectularius* was currently undergoing lineage divergence through host association (Booth et al. 2015, Wawrocka et al. 2015).

Beyond reproductive and host factors, environmental factors such as temperature and other microclimatic conditions may result in species variation and speciation. For Ghana, which is characterized by a tropical climate, seven agroecological zones are known across the country that shows variable temperature and rainfall patterns that have shaped fauna and flora species populations across the region (Asare-Nuamah and Botchway 2019). Evidence shows that each of these ecological zones are inhabited by sibling and cryptic species of *Anopheles* mosquito (Appawu et al. 1994). The contribution of these variable ecological regimes to shaping species population and segregating species populations have been widely reported in *Glossina* spp. (Diptera: Glossinidae) and *Apis* spp. (Hymenoptera: Apidae; Ebhodaghe et al. 2017, Combey et al. 2018). Paleo-environmental reconstructions indicates that historical orographic and climatic changes can cause biogeographical isolated populations, such as in the case of sandflies (Psychodidae; Moo-Llanes et al. 2018).

Variable degrees of anthropogenic disturbance can also promote segregation of populations into separate units leading to the formation of species complexes (Florin et al. 2011). Presently, bed bugs are facing excessive selective pressure through anthropogenic control via insecticide applications, with the recent resurgence. This pressure can alter the genetic and morphological life history of a species. This has been evident in some Hemipteran pest populations, such as *Triatoma infestans* (Hemiptera: Reduviidae), where the insect phenotypic integrity was reported to be affected by sublethal doses of pyrethroid insecticides (Nattero et al. 2021).

When speciated populations emerge from evolutionary forces, individuals carrying novel traits are actively or passively dispersed through anthropogenic and environmental means. These novel traits are passed across generations resulting in species variations that can alter the already existing genetic formation and traits of known species. The existence of speciated subpopulations established in a known species population can pose several challenges especially regarding pest population management that employs behavioral, biological, and insecticide control. In the case of the fall army worm (*Spodoptera frugiperda*; Lepidoptera; Noctuidae), the Nucleo Polyhedrosis virus (NPV) used in biological control of the insect varied with regard to strains and pheromonic divergence within the insect population (Fuxa and Richter 1990, Groot et al. 2008, Unbehend et al. 2013).

Cape Coast is one of Ghana’s most visited historical towns and attracts numerous international visitors each year. Human activities such as increasing international travel and trade, has led to the passive spread of bed bugs across the globe (Doggett et al. 2018), and Ghana is experiencing severe bed bug problems (Deku et al. 2021). Subpopulations of the common bed bugs species, *C. lectularius*, have been identified in Paris, France, a major international tourist destination, through molecular analysis (Chebbah et al. 2021), although limited morphological analyses were undertaken. The existence of distinct subpopulations in multiple populations of a single species could impact control strategies through differences in behavior and responses to physical, mechanical, and chemical control measures as noted above. We tested the hypothesis that morphologically variable species of *C. hemipterus* are present in Cape Coast, a major tourist destination site. To do this, we examined bed bug specimens collected from different communities in Cape Coast to determine if populations could be distinguished using a landmark-based geometric morphometric analysis. We also aimed to provide a morphometric review of the nymphal stages of the bed bugs present with the aim of developing a taxonomic key to identify nymphal stages.

## Materials and Methods

### Bed Bug Sampling and Laboratory Maintenance

**Fig. 1.**
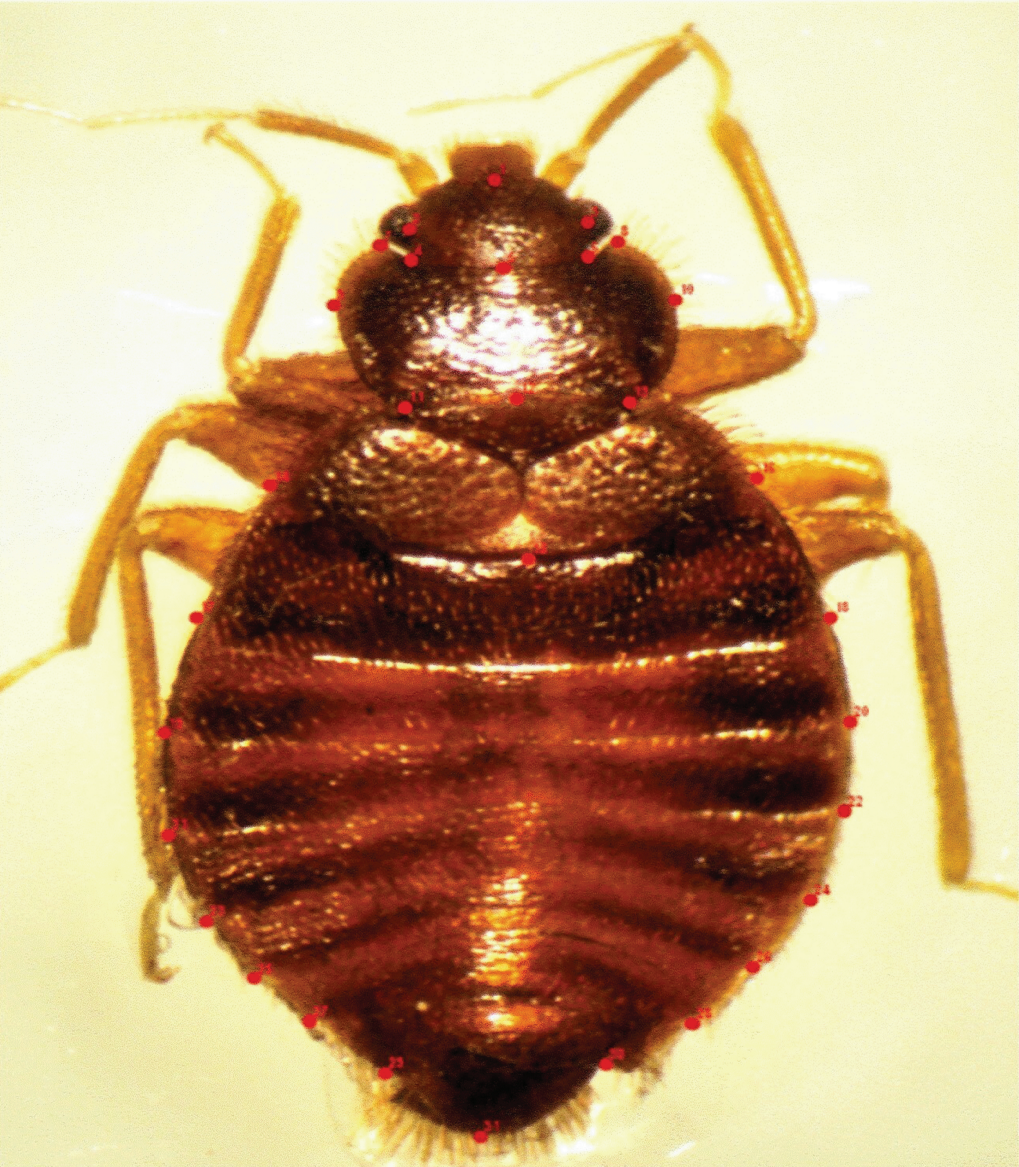
Landmarks manually plotted on the whole body of *Cimex hemipterus.*

Field strains of *C. hemipterus* of different stages (excluding eggs) were collected from nine communities (Figure 1) across Cape Coast Metropolitan District (coordinates: 05°06ʹN 01°15ʹW and 5.1°N 1.25°W, temperature: 24–32°C and relative humidity [RH]: 85–99%). The bed bugs were collected from infested homes and placed into 200 ml plastic containers, transported to the Entomology laboratory, and maintained at 27.1 ± 0.9°C and 75 ± 5% relative humidity. All adult bed bugs were identified as *C. hemipterus* as per the taxonomic keys of Usinger (1966). Colonies of bed bugs obtained from the various locations were fed on an anesthetized rabbit, with rearing conditions of 25.0–27.5°C, and relative humidity of 70–80%. These conditions were maintained throughout the experiment. The newly hatched first nymphal instars obtained from blood-fed females were placed into 200 ml plastic containers and fed on a rabbit according to the protocols of Damodar et al. (1962) throughout the nymphal development.

### Geometric Morphometric Assessment

Landmark-based geometric morphometric examines and compares the relative positions of landmarks between individuals or groups. It focuses on shape variation and is accomplished through the ‘Procrustes paradigm’ (Adams et al. 2013). Geometric morphometric methods have the ability to describe diversity between varying shapes using different sets of statistical parameters. In this study, the geometric morphometric analyses were undertaken following the procedure by Park et al. (2013).

### Specimen Selection for Analyses

A total of 270 field adult bed bugs were used in the analyses (Table 1). Thirty-bed bugs of equivalent number of sexes each from nine populations were used for the geometric assessment.

**Table 1.** Number of adult male and female of *C. hemipterus* assessed from the nine collections (no. of male = 132; no. of female = 138)

Location (community)	Number of males	Number of females
Brofoyedur	11	19
Ayidan	17	13
Castle (Victoria Park)	10	20
Ntim	16	14
Pedu	17	13
Newsite	12	18
Oldsite	18	12
Coronation	16	14
Adisadel	15	15

### Slide Preparation and Mounting

The 2700 adult bed bug samples were placed into 70% alcohol and then mounted onto separate graticule slides (10 points on slide = 1 mm). The whole body of the adult bed bug was mounted dorsal side up, as observed under a microscope.

### Image Acquisition and Creation of TPS Utility File

Dorsal body images of the 270 adult bed bugs were photographed with a digital camera coupled to a stereomicroscope (BEL Photonics STM PRO; BEL Engineering, INV 100, Monza Italy) using the Leica application suite version 3.4.1. All bed bug samples were placed in a single position such that the main morphological features used in the analyses were clearly visible. Samples were handled carefully to avoid distortion in shape. Specimen and camera distances were kept constant for all specimens, and the same camera settings were employed throughout the study to prevent the introduction of any bias. Image files were saved with a specific identifiable name, in low compressed JPEG format. One TPS file was created for all the image files using TPS Util (version 1.49) that served as an input file in TPS Dig2 version 2.31(Rohlf 2015). The TPS file created was then loaded to TPS Dig2 for digitalization.

### Acquisition of Landmarks

Thirty-one anatomically homologous landmarks that are commonly used in beetles (Coleoptera: Carabidae; Lemic et al. 2016, Kang et al. 2020) were manually plotted on the whole body of the bed bug to represent the external body shape of the insect (Figure 1). The TPS Dig2 utility software uses these anatomical points on the head, pronotum, and abdomen of the bed bug to generate Cartesian coordinates (Park et al. 2013).

### Traditional Morphometric Characterization of the Life Stages of *C. hemipterus*

The different life-cycle stages of the bed bugs were morphometrically assessed in the laboratory for body part sizes such as the length and width of the head, pronotum, abdomen, legs, and antennae. Between five and ten (5–10) of the nymphal stages were preserved in 70% ethanol, and mounted on calibrated graticules slides (10 points on slide = 1 mm) for microscopy using a stereomicroscope. The laboratory bed bugs were unfed for 3 day before preservation to reduce the effect of blood meal on the abdomen size. All measurements of the bed bug nymphs were undertaken with the insect held in the dorsal position. Parameters measured (in mm) of the life stages included; Length (L), Abdomen Width (AW), Abdomen Length (AL), Pronota Width (PW), Pronota Length (PL), Head Width (HW), Head Length (HL), Tibial Length (TL), Femoral Length (FL), and Tarsus Length (TsL). Three antennal segments (the last three segments of the antennae were examined, excluding the base or ‘scape’ as preliminary assessment showed that it was too short for assessment); second antennal segment Length (A_2_L), third antennal segment Length (A_3_L), and fourth antennal segment Length (A_4_L).

### Statistical Analysis

#### Geometric Morphometrics Assessment

The digitized data set was analyzed using Morpho J software version 1.06d (Klingenberg 2011). Generalized procrustes analysis was carried out to superimpose landmark coordinates as shape variables through this process and extraneous features (size, orientation, and position effects) are removed (Ross et al. 2010). A preliminary analysis was undertaken to check for outliers by removing mis-digitized specimens. Classifiers used in the MorphoJ were community and sex. Analyses were performed differently for the different sex populations and between sex populations to eliminate the effect of sexual dimorphism. Procrustes ANOVA was performed to estimate the error introduced by imaging and digitizing (Klingenberg 2015). Principal component analysis (PCA), Canonical variate analysis and Mahalanobis distance were calculated by 10,000 random permutations to quantify morphological differences in the populations. PCA was performed to determine the main species composition by differentiating the bed bug populations from the different locations in terms of shape morphometrics. PCA simplifies data set and makes it easier to interpret findings by revealing sub and major groupings of the different populations. Eigenvalues of the PCA represent factors that contribute to variations and only Eigenvalues greater than one should be considered as per Kaiser (1960). The scatter plot of the PCA shows clusters and overlaps of groups based on similarity and in this case was used to explain morphological similarity in the bed bug populations. Canonical variate analysis (CVA) also demonstrates differentiation and reveals the level of morphological similarity and extent of dispersion. CVA determines whether predefined groups can be statistically distinguished based on multivariate data. Each Canonical variates (CVs) obtained from CVA is oriented to summarize the maximal difference among groups. In this case, it was used to reveal variability between the bed bug populations. Ninety-five percent mean confidence eclipses were employed to explain extent of overlap within populations. Discriminant function analysis (DFA) is reliable at predicting group membership and effective at sexing population by revealing sexual dimorphism. The discriminant function test was applied to complement the CVA in distinguishing the population and sexes. Hotelling’s *T*^2^ (Multivariate *t*-test) and Procrustes distances are products from DFA. For the DFA, 1,000 random permutations test was applied to quantify morphological differentiation between the bed bug populations. The preliminary Procrustes superimposition was meant to remove size-related information, however the data set still contains some size-related information, thus the allometric relationship between size and shape are important (Klingenberg 2016). Size and allometry correction in multiple groups usually employs a regression of shape on centroid size or regression of shape on log-transformed centroid size. Centroid size is the square root of the sum of squared distances of all the landmarks of an object from their centroid (center of gravity obtained by averaging X and Y coordinates). Centroid size analysis was performed in SPSS version 21.0. Regression analysis was applied to correct the effects of size on shape. The residuals from the regression analysis are shape values from which the influence of size have been eliminated (Klingenberg 2016). In this study, a regression of Procrustes coordinates as the dependent variable against centroid sizes was assessed. The principal component was also regressed against centroid sizes (pooled within multiple groups). In all cases, 10,000 random permutation tests against the null hypothesis of independence were used.

## Results

### Geometric Morphometrics

The results of the multivariate analysis of variances undertaken on shape variables of the nine *C. hemipterus* populations collected from different communities in Cape Coast indicated a general segregation in the populations. The main patterns of the shape variations were in the relative positions of landmarks associated with the head, pronotum, and in the abdomen.

### Procrustes ANOVA

Procrustes ANOVA revealed that the mean square of interpopulation (individual) variations exceeded the measurement error (Table 2); hence, Procrustes distances can therefore be considered as valid variables for testing significance between and among groups.

**Table 2.** Procrustes ANOVA of *C. hemipterus* bed bug populations from nine locations in Cape Coast, Ghana showing that mean square of individual variation was significant (*P* < 0.0001) from the measurement error in both sex populations

Male						
Effect	SS	MS	df	*F*	*P*-value	Pillai tr.
Individual	0.038	0.0000725	522	2.35	<0.0001	5.15
Residual	0.22	0.000030	7018			
Female						
Effect	SS	MS	df	*F*	*P*-value	Pillai tr.
Individual	0.04	0.000077	522	1.81	<0.0001	5.24
Residual	0.30	0.000042	7018			

SS = sums of squares; MS = mean of sums squares; df = degree of freedom; *F*-value of *F* test.

### Principal Component Analysis

**Fig. 2.**
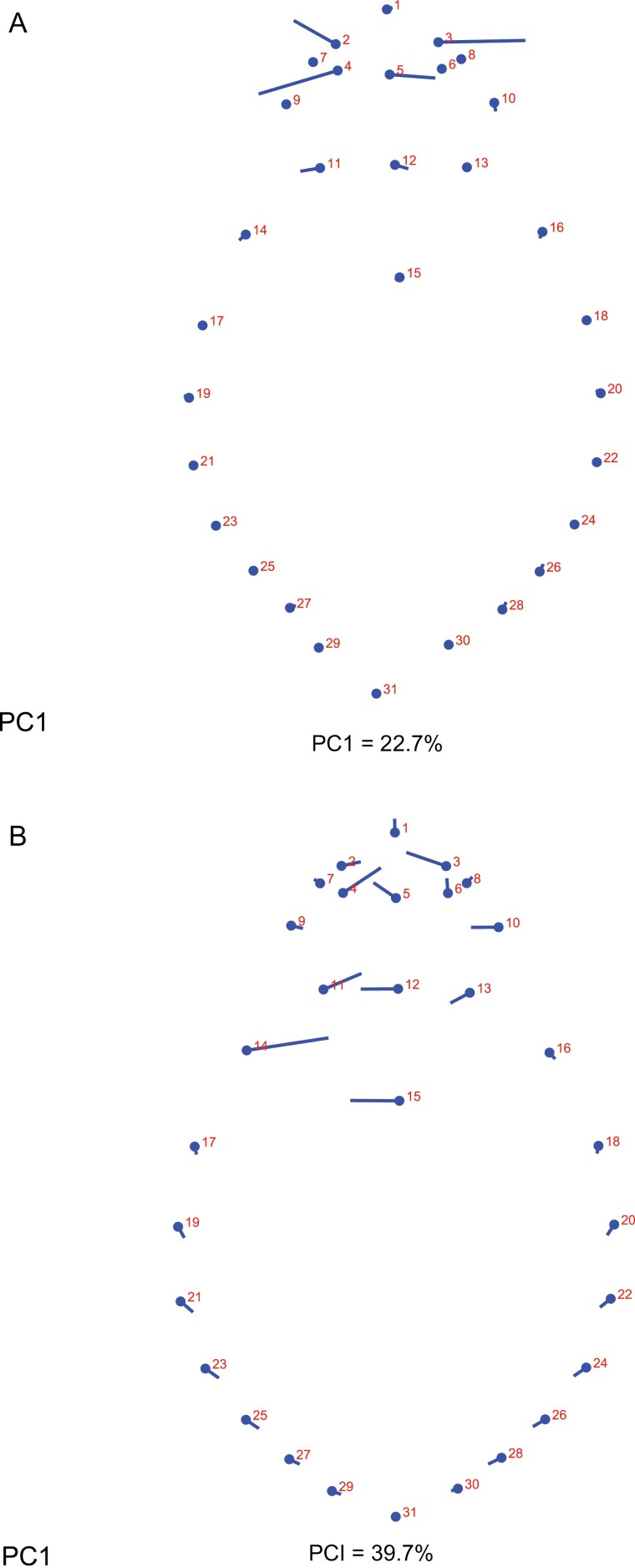
Shape changes (PC1) during principal component analysis of male (A) and female (B) populations of *C. hemipterus* from Cape Coast showing changes of landmarks located on the pronotum and head.

**Fig. 3.**
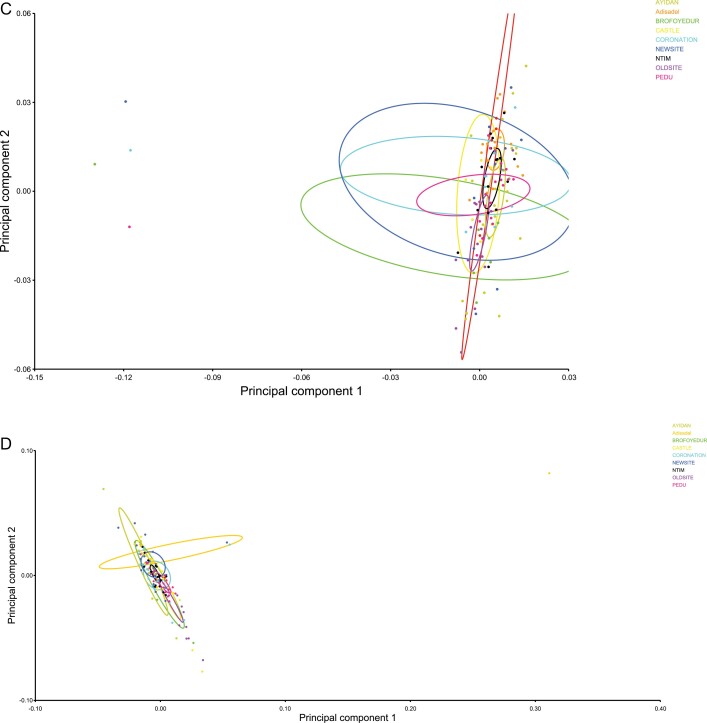
Scatterplot of the major principal components (PC2 vs PC1) which accounted for 42.7% (PC1 + PC2) and 57.2% (PC1 + PC2) variability respectively, in male (C) and female (D) showing clustering and segregation occurring in the nine *C. hemipterus* populations from Cape Coast.

PCA showed variability among the nine *C. hemipterus* populations. For male populations, PCA showed that variances of the first 16 Eigenvalues were >1% and contributed 86.8% of data variability. The % variances of the Eigenvalues represent various proportions of the principal components (PCs; shape variables) that contributed significantly to the variations among the populations. PC1 is the first principal component and the major shape variable contributing to variations among the populations. PC1 (22.7%), PC2 (19.9%), and PC3 contributed cumulatively 52.9% to the variation. Shape changes associated with the principal components contributing variations in males revealed that PC1 suggested variations in relative positions of landmarks 2, 3, 4, 5, 11, and 12 (out of a total 27 landmarks plotted) associated with the head and pronotum (Figure 2). In the females, PCA showed that variances of the first 12 Eigenvalues were >1% and contributed 89.4% of the data variability. PC1 (39.4%) and PC2 contributed cumulatively 57.2% to the variation. PC1 of females constituted variations in relative positions of landmarks 1, 2, 3, 4, 5, 6, 10, 11, 12, 13, 14, and 15 associated mainly with the head and pronotum and part of the first abdominal tergites (Figure 2). The scatter plot using PC2 against PC1 revealed clustering and overlaps of most of the population members in both sexes. However, subgroups were present within the clustered populations (Figure 3).

### Canonical Variate Analysis and Discriminant Function Analysis

**Fig. 4.**
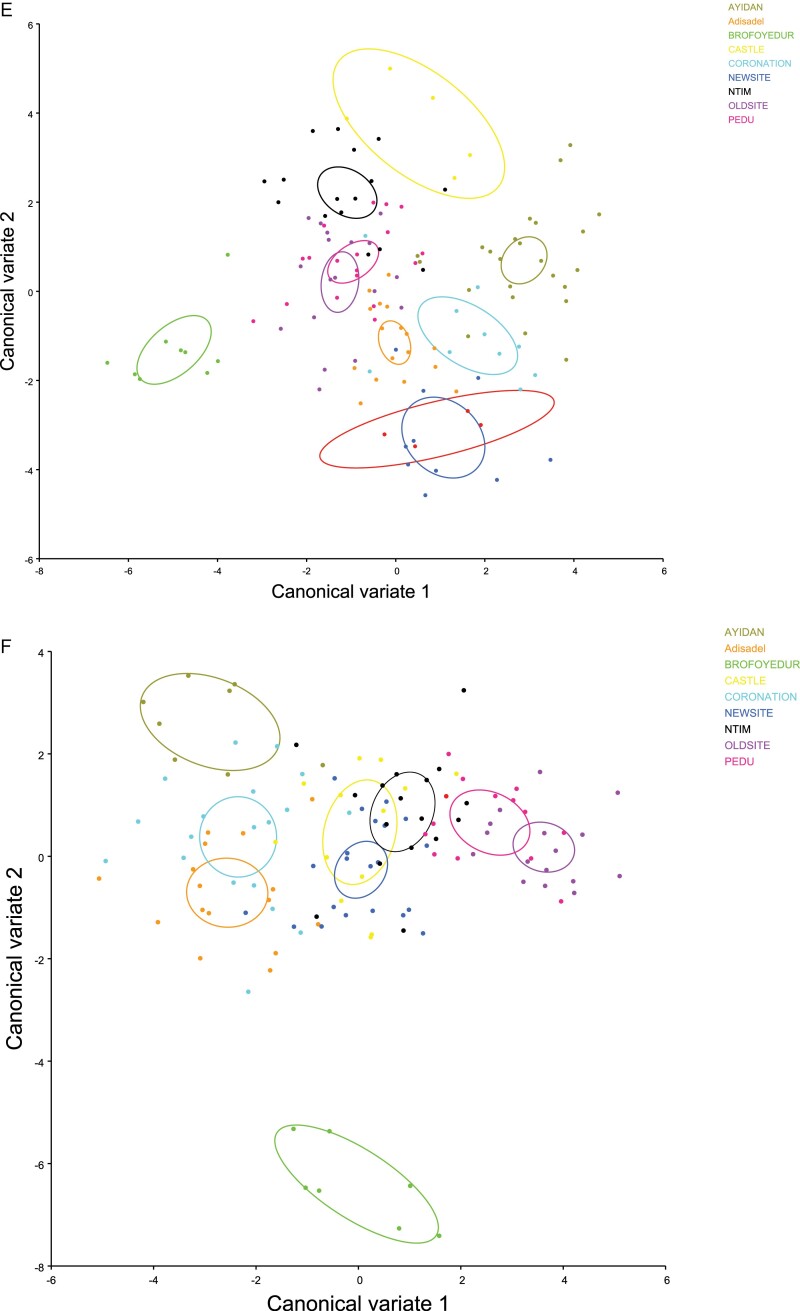
Canonical variate analysis (CVA) of body shape of male (E) and female (F) *C. hemipterus* from Cape Coast: Scatterplot of the major canonical variates (CV2 against CV1) (D) which accounted for 45.9% (CV1 + CV2) and 48.3% (CV1 + CV2) respectively in males and females showed clustering and segregation occurring in the nine *C. hemipterus* populations. The 95% mean confidence eclipse. Different colors represent the various populations (See online version for color figure).

Canonical variate analysis revealed several subgroupings among the nine *C. hemipterus* populations although there was an overlap mainly in females due to high dispersion. For both sex populations, CVA showed nine Eigen factors that contributed 100% of data variability. The major contributors to the variations were characters represented by CV1 and CV2 contributing 27.2% and 18.7%, respectively to the variation in males. Whereas, in the females, CV1 and CV2 contributed approximately 29.4% and 18.9%, respectively. In both sex populations, a scatter plot using CV1 versus CV2 showed differentiation among the nine-bed bug populations according to 95% mean confidence eclipses resulting in several subgroupings and few overlaps between some populations commonly in females due to high dispersion (Figure 4). Permutation tests run over Mahalanobis distance matrices showed significant differences (*P* < 0.0001) between group pairs of male populations for all communities except between parings of Castle with Ayidan and Brofoyedur and Coronation and between Pedu and Oldsite. For females, significant differences (*P* < 0.0001) existed between all population’s pairings from the nine communities, except group paring of Ayidan and Brofoyedur.

**Fig. 5.**
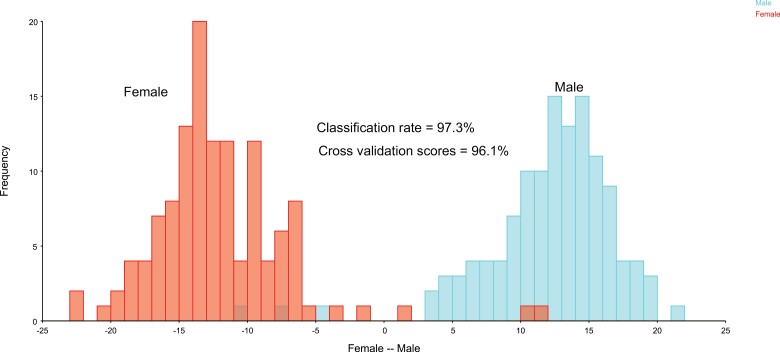
Discriminant function analysis (DFA) test for populations of male and female *C. hemipterus* from Cape Coast, Ghana. Permutation test (10,000 permutation rounds) for Mahalanobis distances showed differences between the sexes (*P* < 0.0001). Male’s graph indicated with blue color on right. Female’s graph indicated with red color on left (See online version for color figure).

**Fig. 6.**
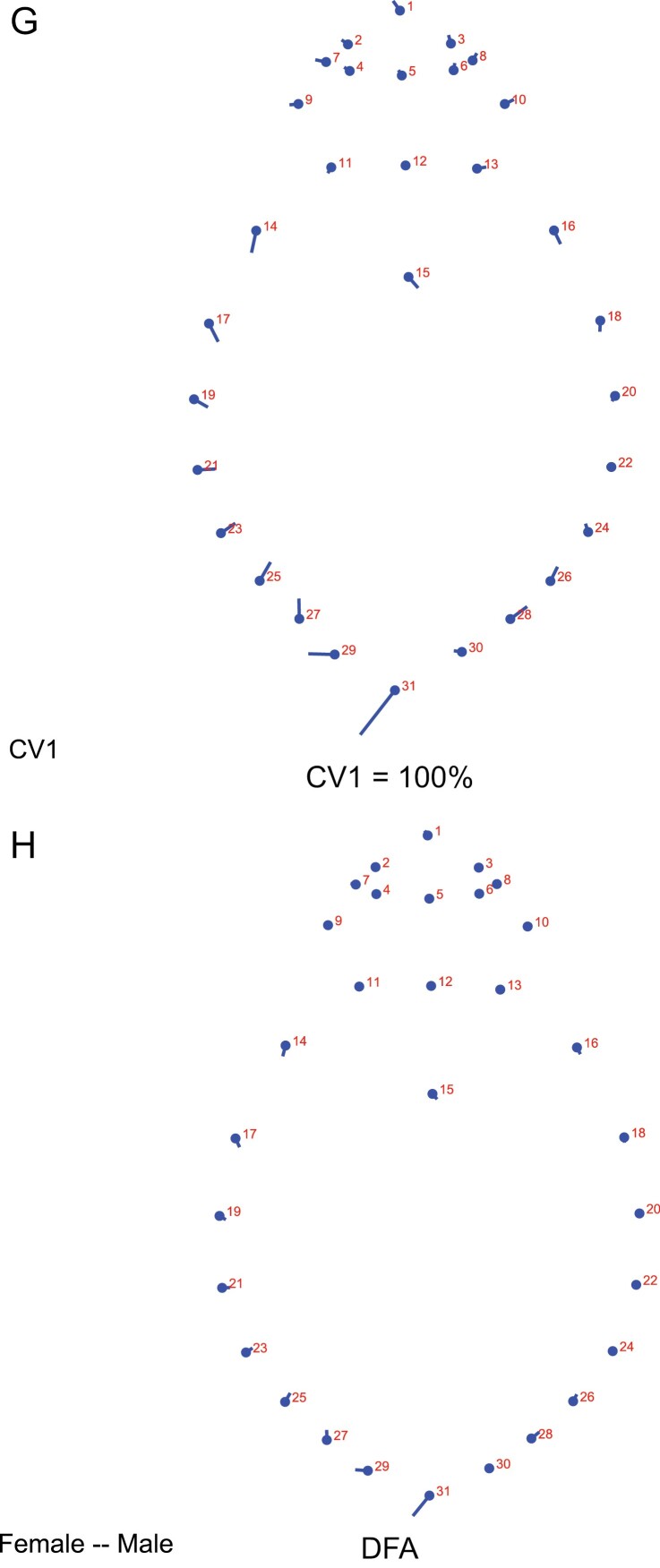
Shape changes of the major canonical variate (CV1) (G) and discriminant function analysis (DFA) test (H) for *C. hemipterus* males and females from Cape Coast showing changes in landmarks associated with the abdomen.

Between the sexes, a strong sexual dimorphism for shape was evident. CVA showed 100% differentiation between male and female populations similarly as DFA (Figure 5). Shape changes showed that Landmarks contributing to variations were associated mainly with the abdomen including the landmark located on the male genitalia on the last abdominal segment as contributing mainly to the variations between sexes (Figure 6). Landmarks on the pronotum and head did not contribute significantly to the variations between the sexes unlike variations between populations (Figure 6). Permutation tests run over Mahalanobis distance matrices showed significant differences between the sexes (Mehalanobis distance = 4.8, *P* < 0.0001) as well as Procrustes distance (Procrustes distance = 0.03, *P* < 0.0001).

Discriminant function analysis test distinguished between pairwise groupings of some populations with high classification rates and cross-validation scores after 1,000 random permutations, indicating the presence of segregation occurring within the nine *C. hemipterus* populations (Table 3). However, Castle population was not separated from any of the populations, similarly, Mahalanobis distance evaluation found no significant differences (*P* < 0.001) between group pairings of Castle populations with Brofoyedur and Coronation and Ayidan populations indicating high dispersion levels of Castle populations across communities. DFA distinguished between the sexes with high discriminant and cross-validation scores (Figure 5). Shape changes obtained from the DFA for sexes was similar to the shape changes obtained during the CVA with the major deviation in landmark associated with the male genitalia located on the last abdominal segment (Figure 6).

**Table 3.** Discriminant function analysis test showed high mean classification rates and cross validation scores after 1000 random permutations against the null hypothesis: Pairwise groupings of some populations showed statistical significance (*P* < 0.001) in Procrustes distances and Hotelling *T*^2^ indicating the presence of segregation occurring within the nine collections of *C. hemipterus*

Group pairs	Mean classification rate (%)	Mean cross-validation scores (%)	Procrustes distance	Hotelling *T*^2^
Male				
Ayidan–Adisadel	100	74.9	*P* = 0.003	*P* < 0.0001
Ayidan–Ntim	100	95.0	*P* < 0.001	*P* < 0.0001
Ayidan–Pedu	100	79.1	*P* = 0.005	*P* < 0.0001
Adisadel–Oldsite	100	83.0	*P* < 0.001	*P* < 0.0001
Adisadel–Pedu	100	68.8	*P* < 0.001	*P* < 0.0001
Coronation–Broyedur	100	90.0	*P* = 0.03	*P* < 0.0001
Adisadel–Ntim	100	73.2	*P* < 0.001	*P* < 0.0001
Brofeyedur–Ntim	100	76.4	*P* < 0.0001	*P* < 0.0001
Coronation–Ntim	100	88.8	*P* = 0.005	*P* < 0.0001
Coronation–Oldsite	92.2	63.3	*P* < 0.001	*P* < 0.0001
Brofeyedur–Oldsite	92.2	85.9	*P* < 0.001	*P* < 0.0001
Adisadel–Brofoyedur	100	97.0	*P* < 0.001	*P* < 0.0001
Female				
Adisadel–Oldsite	100	90.6	*P* < 0.0001	*P* < 0.0001
Adisadel–Pedu	100	85.4	*P* = 0.03	*P* < 0.0001
Brofoyedur–Ntim	100	78.6	*P* < 0.008	*P* < 0.0001
Brofoyedur–Pedu	100	78.6	*P* = 0.002	*P* < 0.0001
Newsite–Oldsite	97.61	62.9	*P* < 0.0001	*P* < 0.0001
Ntim–Oldsite	94	69.9	*P* < 0.0001	*P* = 112

Hotelling *T*^2^**=** multivariate *t*-test.

### Centroid Size Evaluation and Regression Analysis

Centroid size analysis showed statistical significances among both sex populations (female [*F* = 53.61, df = 8, *P* < 0.0001], male [*F* = 38.6, df = 8, *P* < 0.0001]), which suggests that males and females of some populations are larger in terms of size. Centroid size differs between the sexes (*P* < 0.05, [female mean = 2,512.35 ± 306], [male mean = 2,318.45 ± 372.12]) indicating that females were significantly larger than males in size. Multivariate regression analysis of shape variables against centroid size meant to correct size indicated minimal correlation. The allometric effect of size on shape in both sex populations was very minimal. Although the regression of Procrustes coordinates versus centroid sizes was significant (*P* < 0.0001) in both sexes, the contribution of the allometric percentages were negligible (Table 4). Moreover, separate regression of the most important shape variable (PC1) against centroid size in both sexes was not statistically significant (*P* > 0.0001; Table 4). In all cases, 10,000 random permutations against null hypothesis of independence were used.

**Table 4.** Multivariate regression analysis of shape variables against centroid size: Procrustes coordinates versus centroid size and PC1 versus centroid sizes of the nine *C. hemipterus* groups from Cape Coast, Ghana pooled within group showed statistical significance in the Procrustes coordinates against Centroid size (*P* < 0.0001) after 10,000 random permutations

Procrustes coordinates versus Centroid size				
Male				
%Predicted	Total SS	Predicted SS	Residual SS	*P*-value
4.5%	0.22	0.0098	0.21	<0.0001
Female				
%Predicted	Total SS	Predicted SS	Residual SS	*P*-value
5.44%	0.30	0.016	0.28	<0.0001
PC1 versus Centroid size				
Male				
%Predicted	Total SS	Predicted SS	Residual SS	*P*-value
0.95%	0.064	0.0006	0.063	0.21
Female				
%Predicted	Total SS	Predicted SS	Residual SS	*P*-value
2.58%	0.120	0.003	0.115	0.079

SS = sums of squares.

### Traditional Morphometric Characterization of Life Stages of *C. hemipterus* Nymphs

Antennal length (last three segment) and pronotum width (first–fifth instar) measurements are listed in Table 5. Generally, nymphal antennal length (last three segments) and pronotum width are distinctive for each nymphal stage, and progressively increase as they grow.

**Table 5.** Length of antennae (last three segment) and pronota width (PW) of *C. hemipterus* life stages: seven (7–10) each of the life stages (10 points on the graticule slide = 1 mm)

	Antennal length (mm; last three segment)		Pronota width (mm)	
Stage (*n* = 7–10)	Range	Mean	Range	Mean
First instar	0.60	0.60 ± 0.00	0.35–0.40	0.39 ± 0.02
Second instar	0.75–0.80	0.78 ± 0.03	0.45–0.50	0.49 ± 0.02
Third instar	0.90–1.00	9.50 ± 0.04	0.60	0.60 ± 0.00
Fourth instar	1.10	1.10 ± 0.00	0.80–0.90	0.83 ± 0.04
Fifth instar	1.35–1.40	1.35 ± 0.04	1.03–1.1	1.03 ± 0.02

#### First Instar

In the first instars, antennal segments were unequal in length; second (0.10 mm) < third (0.20 mm) < fourth (0.30 mm) segment. The last antennal segment of the first-instar overlaps that of the second instar. Length of the last three antennal segments is detailed in Table 2.

#### Second Instar

Antennal segments (the last three antennal segments) were on average, unequal. The second segment (exactly 0.20 mm) overlapped with the third, but generally was shorter than the third (range: 2.00–0.30 mm; average 2.75mm). Similarly, the third overlapped with the fourth, but was generally shorter than the fourth (range: 0.30–0.35 mm). The length of the second antennal segment in the second instar is exactly twice that of the first instar. The length of the last antennal segment overlapped the first, and third nymph instars.

#### Third Instar

In the third instars, the antennal segment was unequal; second antennal segment (0.25–3.00mm), third (0.30–0.50mm), and fourth (0.35–0.40 mm).

#### Fourth Instar

The second, third, and fourth antennal segments were 0.30 (±0.00), 0.40 (±0.00), and 0.37 (0.35–0.40), respectively. The second antennal segment was distinctively shorter than third and fourth. Both third and fourth antennal segments were subequal.

#### Fifth Instar

The length of the second and third antennal segments ranged 0.45–0.50 mm, and that of the fourth antennal segment was exactly 4.00 mm.

## Discussion

Climate is a major determinant of insect species distribution and abundance (Stange and Ayres 2010). In our research, only *C. hemipterus* was identified in Cape Coast, suggesting that the climatic conditions characterizing the area was preferentially favorable for the survival of this species (Usinger 1966). *Cimex hemipterus* has been reported from many parts of the world within the latitudes of 30° north and south of the equator (Usinger 1966, Doggett et al. 2018), and more recently, outside this range as well (Masini et al. 2020, Balvín et al. 2021). In Africa, *C. hemipterus* has been recorded in many countries, including; Zimbabwe, Tanzania, Nigeria, Ethiopia, Ghana, and Sierra Leone (Fourie and Crafford 2018, Deku et al. 2021), which are characterized by similar climatic conditions.

From our investigations, traditional morphological evidence suggested the presence of similar morpho-species of *C. hemipterus* characterized the geographical location. Geometric morphometry provided evidence to show the presence of morphologically variable specimens of *C. hemipterus* within the study area that varied in shape. The significant characters that contributed to the variations within the multiple populations of *C. hemipterus* are the insect pronotum, the head, and parts of the abdomen. The pronotum is the most relevant taxonomic character within the members of the Cimicidae (Usinger 1966). Both *C. lectularius* and *C. hemipterus* are similar, and commonly distinguished by the presence of an upturned lateral flange on the margin of the pronotum of *C. lectularius* (Ghauri 1973), which makes the thorax of *C. lectularius* wider than that of the *C. hemipterus*. It is possible to suspect that sibling species (identical species that have not been differentiated taxonomically) of *C. hemipterus* might be present in the region and not reported due to limited bed bug research from the country. Between the sexes, there were almost no variations in landmarks associated with the pronotum probably indicating the presence of similar morpho-species. However, sexual dimorphism was detected in the insect abdomen. Both sexual size dimorphism (SSD) and sexual shape dimorphism (SShD) are common phenomenon in insect sex populations (Benítez 2013) and have been reported in bed bugs (Usinger 1966). In the Chilean magnificent beetle (*Ceroglossus chilensis* E.; Carabidae: Coleoptera), shape dimorphism was observed in the insect abdomen attributed to the presence of eggs in the female abdomen (Benítez 2013). The variation in shape of abdomen of male and female bed bug with males having their genitalia on the last abdominal segment is a known morphological character that contributes to sexual dimorphism in bed bugs and are commonly used to sex bed bug species morphologically (Usinger 1966, Saari et al. 2018).

In Africa, an increase in bed bug infestations has been very consistent even during the 1950s to the 20th century when elsewhere there was a reduction in global bed bug trends (Boase 2001, Potter 2011, Doggett et al. 2018). Socioeconomic problems and insecticide resistance are contributing to the increasing bed bug infestation issues in Africa (Fourie and Crafford 2018, Deku et al. 2021), while inadequate bed bug research could be compounding the problem. In our present study, it was shown for the first time through morphometric surveys, the possible presence of subpopulations established within multiple populations in the region. Recently, genetically variable populations of *C. lectularius* were detected from Paris, France with limited morphological evidence (Chebbah et al. 2021). The presence of subpopulations established in multiple populations of *C. hemipterus* could have serious insecticide control implications. To date, 12 knockdown resistance mutations against pyrethroids have so far been detected in *C. hemipterus*, (as against three detected in the *C. lectularius*; Punchihewa et al. 2019, Dang et al. 2021). Multiple populations of *C. hemipterus* exhibiting numerous mechanisms conferring broad spectrum resistance have also been reported (Dang et al. 2021) and recently, the common substitution mutation F348Y conferring organophosphate resistance was detected in *C. hemipterus* populations (Komagata et al. 2021). This suggests the probable presence and widespread diverse insecticide resistance mechanisms in *C. hemipterus* populations in Ghana. As evident in our previous report from Cape Coast, we showed through insecticide bioassays, the reduced susceptibility of *C. hemipterus* populations to both pyrethroids and organophosphate’s formulations (Deku et al. 2021).

Cape Coast is one of the six major Metropolis in Ghana that also provides tourism services to local and international visitors. International travel and trade have been linked to the transfer of bed bugs across the globe and an important factor in the current bed bug resurgence (Doggett et al. 2018). Recently, in a British media publication captioned ‘*A British Airways Flight Was Ground Due to Bed Bugs,*’ a flight from London to Ghana was grounded after the cabin crew refused to fly the plane due to a bed bug infestation in the plane. This is not the first time British Airways has suffered from bed bug infestations (Minton 2018). *Cimex hemipterus* has been reported in several countries for the first time, Russia, Italy, Hawaii, Australia in Florida after about 60 years of disappearance and in several other European countries (Campbell et al. 2016, Gapon 2016, Doggett et al. 2018, Lewis et al. 2020, Chebbah 2021). Subpopulations that showed restricted distribution presents a possible reproductive barrier that could disrupt mating and gene flow between populations. There is an indication of increasing human dispersion and transfer of bed bug populations across and between communities showing overlap due to closeness in the geographical proximity between locations (Deku et al. 2021), suggesting also that bed bugs from widely spread or overlapping communities can interbreed.

Subpopulations may have an impact on nonchemical control measures (physical and mechanical) as variable populations could display differences in behavior and responses to intraspecific chemicals used as an attractant in lures. Pheromonic divergence has been detected in the fall army (*Spodoptera* sp.) due to the presence of subpopulations within the insect population (Groot et al. 2008, Unbehend et al. 2013). Plus, there are reports on strains of the pest responding differently to NPVs (Fuxa and Richter 1990). What is more fascinating is that, Cape Coast provides tourism services and attracts international and local visitors across the world. The possibility of the city becoming host to variable bed bug strains with diverse behavior and even variable insecticide resistance genes, that could become a source to other geographical locations across the world may occur.

With modifications in general body shape, taxonomic implications are expected as the species can easily be mistaken for the sister species, *C. lectularius*, and other related members of the insect family. Morphologically, it can be challenging when separating the two major human-dependent bed bug species; hence, this morphological adaptation may contribute to further confusion in distinguishing the two species. Variable ecological and reproductive implications such as moveable adaptive behaviors, disruption of mate selection, and gene flow are also expected. In *C. lectularius*, two genetically and morphologically distinct lineages were identified, and crosses between the different host lineages were unsuccessful, which was attributed to the presence of postmating barriers and reproductive isolation between lineages (Wawrocka et al. 2015).

The emergence of possible subpopulations of *C. hemipterus* could be attributed to multiple factors that results in sympatric speciation in the insect species. These include sexual conflicts (Engen and Bernt-Erik 1985, Gavrilets and Waxman 2002), host factors (Booth et al. 2015, Wawrocka et al. 2015), climate change, and variations in environmental conditions (Moo-Llanes et al. 2018). It is possible that the variable ecological zones with different physical conditions across the country could lead to the morphological differentiation in the tropical bed bugs as these variable climatic conditions in the various ecological zones in Ghana have produced morphologically variable populations of mosquitoes, testseflies, and bees (Appawu et al. 1994, Ebhodaghe et al. 2017, Combey et al. 2018) Furthermore, environmental isolation resulting in ecotypic morphological diversity is an important phenomenon that occurs in plant, aquatic animal species, and birds (Mayr 1954, Banks 1970, Keeley et al. 2007). This has been reported in insect species including, Assassin bugs, *Acanthaspis quinquespinosa* F. (Hemiptera: Reduviidae; Sahayaraj 2007) and *Rynocoris fuscipes* F. (Hemiptera: Reduviidae; Bascar et al. 2012). Morphological body shape may also indicate the impact of environmental stressors on the insect population (Sommer and Nielsen 1992, Alves and Hernández 2017). It is also possible that anthropogenic environmental stressors in the communities, such as the excessive application and prolong use of insecticides by households to control bed bugs in the study area as evidenced in our previous investigations (Deku et al. 2021), may introduce environmental pressures resulting in morphological shape differentiation. Recently, it has been reported that, phenotypic integrity of populations of *Triatoma infestans* were affected by sublethal doses of pyrethroid insecticides (Nattero et al. 2021). However, it is not presently known if this has contributed to the observed body shape variability in *C. hemipterus* and if there are implications for control, or why insecticide selection pressure would lead to changes in body shape. Modification in body shape might have also evolved from survival adaptations as changes in general body shape in some ectoparasites facilitate movement on the host and enable them to hide in tight spaces (Burkett-Cadena 2019). At this stage, we have no information on the significance of the morphological differences in general body shape in the subpopulations of *C. hemipterus* in relation to management strategies. This will be the basis of further investigations.

There is no nymphal key for *C. hemipterus* in the *Monograph of the Cimicidae* (Usinger 1966). A possible key for separating the nymphs has been proposed by Newberry (1990) for *C. hemipterus* collected in Natal, South Africa, using pronota bristles and head width size. To the best of our knowledge, there has not been any published work that has successfully employed this key in separating the nymphal stages, and these identification keys are posed with complexities of counting the nymphal pronotum bristles, which can be extremely difficult to count and often lost on bed bugs. Findings from the nymphal antennal characterization are hereby outlined below that could be used as a tentative nymphal supplementary morphological key.

### Morphometric Guide to the Identification of Nymphal Stages of *Cimex hemipterus* in Cape Coast, Ghana

**Note:** Generally, nymphal antennal length (last three segments) and pronota width are morphometrically distinct for each nymphal stage, and progressively increase in length with later instars.

I. Key to first-instar nymph
  1. Last three antennal segments (second, third, and fourth) unequal.
  2. Second antennal segment shorter than third, and third shorter than fourth.
  3. Fourth antennal segment as long as length of second plus third.
ll. Key to second-instar nymph
  1. Length of second antennal segment twice that of the first instar.
  2. Fourth antennal segment shorter than the second plus third.
III. Key to third-instar nymph
  1. Length of fourth antennal segment generally greater but overlap the third antennal segment.
IV. Key to fourth-instar nymph
  1. Second antennal segment distinctively shorter than third and fourth.
  2. Third and fourth antennal segments subequal.
V. Key to fifth-instar nymph
  1. Length of last three antenna segment as long as tibia of hind leg.

As noted above, Newberry (1990) used head width to separate the nymphal stages of *C. hemipterus*. The overlapping that occurs in the head width sizes of nymphal instars places a limitation on using head width on separating the stages. In resolving these challenges, pronota width and antennal length (last three antennal segments) sizes may be diagnostic. In our findings, pronotum width was found distinctive across the life stages, with an increasing life stage having a progressively increasing pronotum width. Findings from Newberry (1990) indicated that the number of pronota bristles increased significantly across the life stages from first instar to adult, which was probably due to the increase in size of the nymphal pronota width, which contains the pronota bristles. Because of the difficulty in counting pronotum bristles, measuring the size of the pronotum width can be supplemented with estimating the pronotum bristles along the width of the pronotum.

In the study herein, antennae length was found distinctive of the life stage and varied significantly across the nymphal stages, and did not overlap unlike characters such as the nymphal head width, head length, and pronota length. A progressive increase in the number of sensilla on the antennae of *C. hemipterus* nymphal to adult stages have been observed in previous studies (Mendki et al. 2013). In a related investigation that examined the structure and distribution of sensilla on the antenna of *Diaphorina citri* Kuwayama, 1908 (Hemiptera: Leviidae), it was also observed that the length of the insect antennae increased significantly with increasing nymphal stage, as well as the number of antennal sensilla (Zheng et al. 2020). Nymphal pronota width and antennal length sizes (first–fifth) were recorded in the *Monograph of Cimicidae* (Usinger 1966) and pronota width of *C. lectularius* and *C. hemipterus* as per Campbell et al. (2018; Table 6). The full antennal length of *C. hemipterus* was found to be distinctive in size for each of the nymphal stages in these previous studies (Table 6). In our current investigations, only the length of the last three antennal segments were measured, as the first antennal segment was too short for an adequate comparison to be made. Note that the fourth antennal segment of the third instar of *C. hemipterus* in our study was generally greater and rarely overlapped the third antennal segment. This is contrary to what was recorded in the *Monograph of Cimicidae* (Usinger 1966) for *C. lectularius*, where the last antennal segment was distinctively shorter than the third antennal segment. This feature can possibly distinguish the third instar of the two-bed bug species. In Table 6, it can also be observed that the nymphal pronota width sizes for both *C. lectularius* and *C. hemipterus* were distinctive for each of the nymphal stages, progressively increasing with the latter stages. In comparison, the pronota width size recorded by both Usinger (1966) and Campbell et al. (2018) for *C. lectularius* nymphal stages was significantly larger than in the nymphal stages of *C. hemipterus* (Table 5). Note that the pronota of *C. lectularius* is broader than that in the *C. hemipterus*, and this has been evidenced in the nymphal stages of the two species (Usinger 1966, Campbell et al. 2018).

**Table 6.** Antennal length and pronota sizes of *C. lectularius* and *C. hemipterus* as listed from the monograph of the Cimicidae (Usinger 1966) and (Campbell et al. 2018)

Usinger (1966)			Campbell et al. (2018)	
*C. lectularius*	Antennal length (mm)	Pronota width (mm)	*C. hemipterus* pronota width (mm)	*C. lectularius* pronota width (mm; mean for two strains)
First instar	0.77	0.46	―	―
Second instar	0.88	0.63	―	0.65
Third instar	1.10	0.74	0.56 ± 0.01	0.81–0.84 (±0.01)
Fourth instar	1.31	1.00	0.76 ± 0.01	0.91–0.96 (±0.01)
Fifth instar	1.58	1.27	0.94 ± 0.03	1.22–1.31 (±0.02)

In conclusion, we show that subpopulations of *C. hemipterus* were present in Cape Coast, Ghana based on general body shape morphology. This may have taxonomic, genetic, ecological, and control implications. Further work with landmark-based geometric morphometrics on other bed bug species collected in disparate populations may further expand and reveal the utility of landmark geometric morphometric analysis in capturing morphological adaptations and segregation in the insect population. The morphological descriptions of the nymphal stages of *C. hemipterus* allow the establishment of nymphal diagnostic characters for effective identification. The ability to distinguish the nymphal stages is also essential for standardization of life stages in insecticide bioassays.
